# Expanding the mutational spectrum of monogenic hypogonadotropic hypogonadism: novel mutations in *ANOS1* and *FGFR1* genes

**DOI:** 10.1186/s12958-020-0568-6

**Published:** 2020-01-29

**Authors:** Agnieszka Gach, Iwona Pinkier, Maria Szarras-Czapnik, Agata Sakowicz, Lucjusz Jakubowski

**Affiliations:** 1 Department of Genetics, Polish Mother’s Memorial Hospital Research Institute, 281/289 Rzgowska Street, 93-338 Lodz, Poland; 2 Department of Endocrinology and Diabetology, Children’s Memorial Health Institute, Warsaw, Poland; 3 Department of Medical Biotechnology, Medical University of Lodz, Lodz, Poland

**Keywords:** Congenital hypogonadotropic hypogonadism, Kallmann syndrome, *ANOS1* mutations, *FGFR1* mutations, Human reproduction

## Abstract

**Background:**

Congenital hypogonadotropic hypogonadism (CHH) is a rare disease, triggered by defective GnRH secretion, that is usually diagnosed in late adolescence or early adulthood due to the lack of spontaneous pubertal development. To date more than 30 genes have been associated with CHH pathogenesis with X-linked recessive, autosomal dominant, autosomal recessive and oligogenic modes of inheritance. Defective sense of smell is present in about 50–60% of CHH patients and called Kallmann syndrome (KS), in contrast to patients with normal sense of smell referred to as normosmic CHH.

*ANOS1* and *FGFR1* genes are all well established in the pathogenesis of CHH and have been extensively studied in many reported cohorts. Due to rarity and heterogenicity of the condition the mutational spectrum, even in classical CHH genes, have yet to be fully characterized.

**Methods:**

To address this issue we screened for *ANOS1* and *FGFR1* variants in a cohort of 47 unrelated CHH subjects using targeted panel sequencing. All potentially pathogenic variants have been validated with Sanger sequencing.

**Results:**

Sequencing revealed two *ANOS1* and four *FGFR1* mutations in six subjects, of which five are novel and one had been previously reported in CHH. Novel variants include a single base pair deletion c.313delT in exon 3 of *ANOS1,* three missense variants of *FGFR1* predicted to result in the single amino acid substitutions c.331C > T (p.R111C), c.1964 T > C (p.L655P) and c.2167G > A (p.E723K) and a 15 bp deletion c.374_388delTGCCCGCAGACTCCG in exon 4 of *FGFR1*. Based on ACMG–AMP criteria reported variants were assigned to class 5, pathogenic or class 4, likely pathogenic. Protein structural predictions, the rarity of novel variants and amino acid conservation in case of missense substitutions all provide strong evidence that these mutations are highly likely to be deleterious.

**Conclusions:**

Despite the fact that *ANOS1* and *FGFR1* are classical CHH genes and were thoroughly explored in several CHH cohorts we identified new, yet undescribed variants within their sequence. Our results support the genetic complexity of the disorder. The knowledge of the full genetic spectrum of CHH is increasingly important in order to be able to deliver the best personalised medical care to our patients.

## Background

Congenital hypogonadotropic hypogonadism (CHH) is a rare disease with a male predominance that is responsible for the absence of spontaneous puberty and sterility in most of the patients. The disorder is triggered by defective GnRH secretion or action resulting in low serum steroid concentrations with normal levels of gonadotropins or isolated gonadotropin deficiency of varying degrees [1]. CHH can present as isolated or syndromic with several non-reproductive symptoms. Defective sense of smell, anosmia or hyposmia, is present in about 50–60% of CHH patients and called Kallmann Syndrome (KS), in contrast to patients with normal sense of smell referred to as normosmic CHH (nCHH) [2]. In addition to GnRH deficiency other developmental abnormalities have been described in CHH, such as cleft lip or palate, renal agenesis, dental agenesis, ear anomalies, congenital hearing impairment, bimanual synkinesis or skeletal anomalies [1, 2].

CHH is usually diagnosed in late adolescence or early adulthood due to lack of spontaneous pubertal development. In cases of severe GnRH deficiency in male patients the symptoms of micropenis and/or cryptorchidism can be recognised at birth or in early infancy [1]. In milder CHH cases patients have a history of normal pubertal development and present with adult-onset hypogonadotropic hypogonadism [3]. In about 10% of cases a reversal of the phenotype can be observed after discontinuation of hormone therapy [4].

Not only the clinically, but also genetically very heterogeneous disorder of CHH constantly challenges clinicians and researchers in their efforts to understand the complex molecular genetics of nCHH and KS.

**Fig. 1:**
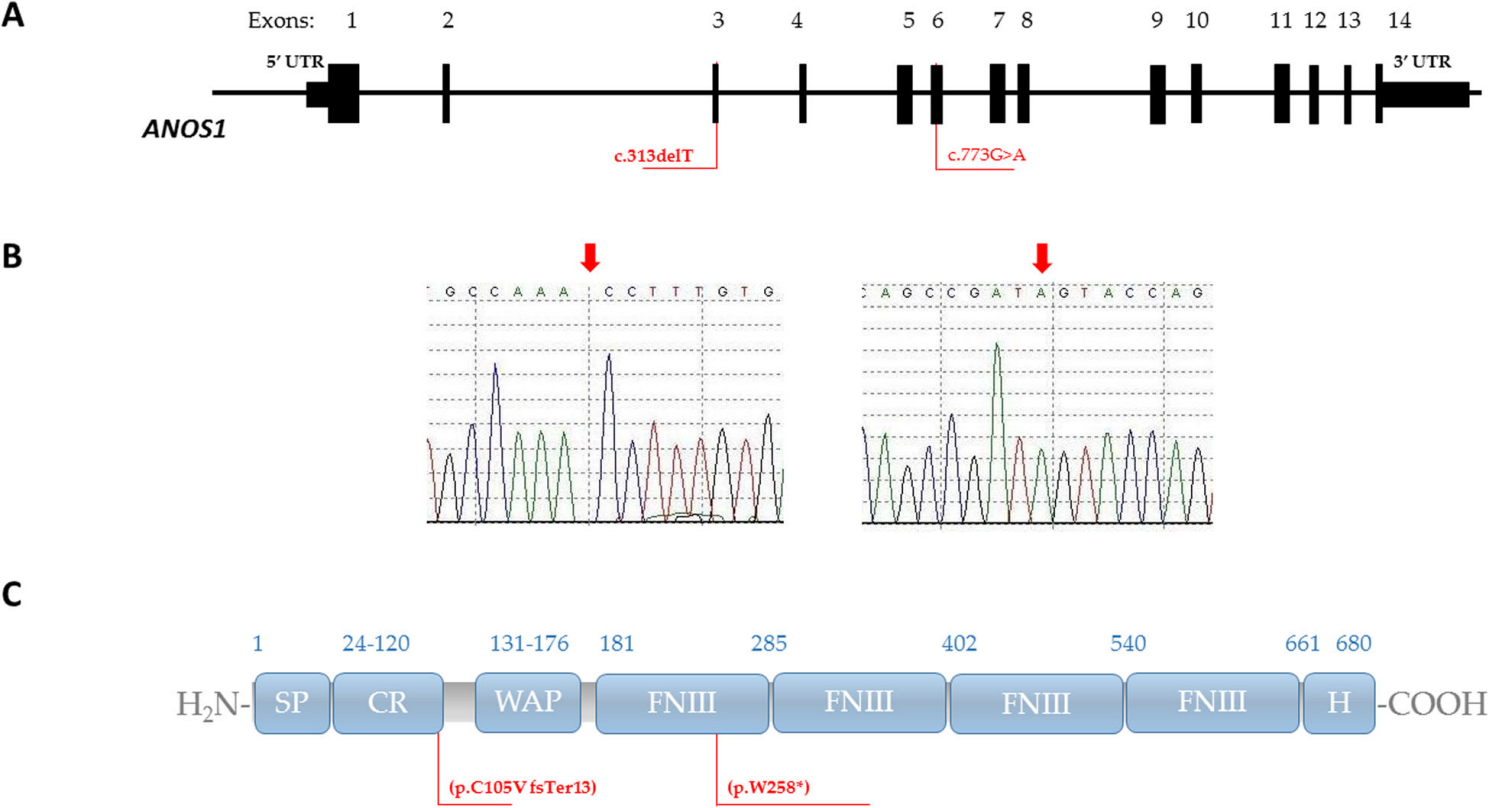
Mapping of *ANOS1* variants to DNA sequence and protein domains. **a** Schematic presentation of *ANOS1* gene, variants’ positions identified in this study are indicated in red. **b** Results of automated DNA sequencing for *ANOS1* mutations in two probands. **c** Schematic presentations of anosmin-1 domains. SP: signal peptide; CR: cysteine-rich region; WAP: whey acidic protein –like domain, FNIII: fibronectin type III domain; H: C-terminal region rich in basic histidine and proline residues; positions of the mutations are indicated in red

*ANOS1* was the first gene linked to Kallmann syndrome pathogenesis [5, 6]. The gene is located on the X chromosome at Xp22.31, contains 14 exons and shows high degree of sequence identity among species. *ANOS1* encodes anosmin-1, a protein which plays an important role in the embryogenesis of brain, kidneys, respiratory and digestive systems [7]. Structurally, anosmin-1 consists of an N-terminal signal peptide, a CR (cysteine-rich) region, a WAP (whey acidic protein-like) four-disulphide core motif and four contiguous FnIII (fibronectinlike type III) domains, followed by a histidine-rich C-terminus (Fig. 1c). This extracellular matrix protein binds to the cell membrane, stimulates axonal outgrowth and acts as an axonal guidance molecule for GnRH neurons, olfactory cells and Purkinje cerebellum neurons [8]. Importance of anosmin-1 in the development of olfactory system and migration of GnRH neurons was demonstrated based on findings from two foetuses, one harbouring *ANOS1* deletion and the other a nonsense *ANOS1* mutation. In both cases the olfactory axons and GnRH neurons left the olfactory placode but accumulated over the cribriform plate failing the migration process [9, 10]. *ANOS1* mutations are identified in 5–10% of KS patients and they seem to consistently impair the sense of smell. Based on The Human Gene Mutation Database more than 150 pathogenic variants have been reported in ANOS1, they include deletion of the entire gene, deletion of one or more exons, deletion of several nucleotides, missense, nonsense, and splice variants. Due to the X-linked transmission the disease affects men, yet females heterozygous for an *ANOS1* pathogenic variant may occasionally display clinical features that are diagnostic of isolated GnRH deficiency [11].

**Fig. 2:**
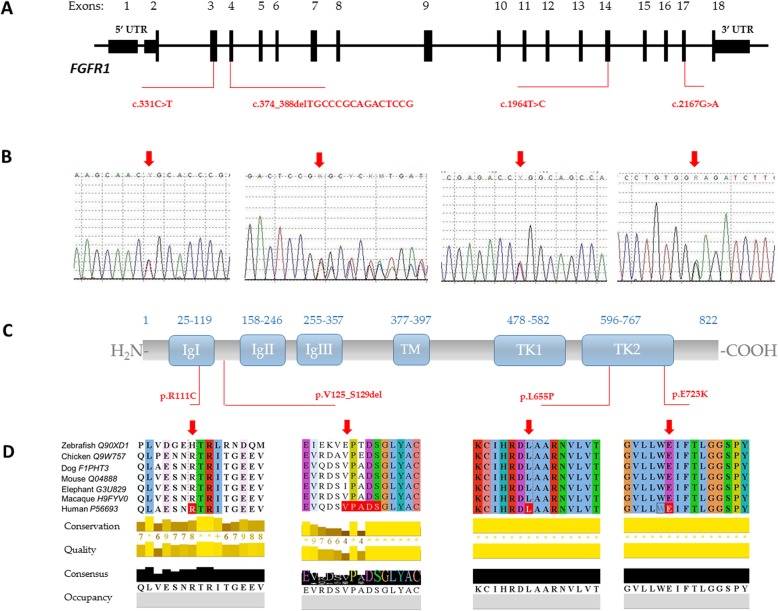
Mapping of *FGFR1* variants to DNA sequence and protein domains. **a** Schematic presentation of *FGFR1* gene, variants’ positions identified in this study are indicated in red. **b** Results of automated DNA sequencing for *FGFR1* mutations in four probands. **c** Schematic presentations of FGFR-1: IgI, IgII and IgIII: three immunoglobulin-like domains; TM: a transmembrane helix; TK1, TK2: two intracellular domains; positions of the mutations are indicated in red. **d** UniProt alignment of FGFR-1 regions containing variants identified in this study and amino acid variations across species from zebrafish to human

*FGFR1* is located at 8p.11.2 and encodes for type 1 fibroblast growth factor (FGF) receptor. FGFR-1 is a member of the tyrosine kinase superfamily of receptors. The receptor contains an extracellular domain that has three immunoglobulin-like domains (IgI, IgII and IgIII) responsible for the receptors affinity and specificity for its ligands. It also includes a single transmembrane helix and two intracellular domains (TK1, TK2) with tyrosine kinase activity (Fig. 2c). FGFR-1 signalling through MAPK pathway is crucial for neuronal migration, differentiation, and survival, as well as cell proliferation during embryonic development [12, 13]. Mice with loss-of-function *Fgfr1* mutations show markedly reduced amount of GnRH neurons [14]. First report on *FGFR1* mutations in KS phenotype was published in 2003 documenting four familial and eight sporadic cases [15].

Heterozygous *FGFR1* mutations are found in 10% of KS and in 6% of all CHH individuals [16]. Pathogenic variants in *FGFR1* include missense, nonsense, splice variants and in rare cases deletions and cause both KS and normosmic CHH with autosomal dominant mode of inheritance. They are linked to highly variable phenotypes, ranging from isolated hyposmia, delayed puberty to severe form of CHH with non-reproductive anomalies [15, 17].

It has been almost 30 years, since the first gene *ANOS1/ KAL1* associated with KS was identified and despite advanced high-throughput technologies causative mutation can be found in less than half of the cases. Genes, whose mutations have been implicated in CHH, are necessary for proper GnRH neuron development/migration, GnRH secretion or pituitary response and function. To date more than 30 genes have been linked to CHH pathogenesis with X-linked recessive, autosomal dominant, autosomal recessive and oligogenic modes of inheritance [18, 19]. Monogenic cases are either sporadic or familial, while genetic variants in non-Mendelian oligogenic cases are most often of parental origin. The genetic architecture of CHH is further complicated by highly variable penetrance of some causative mutations and other genetic and environmental modulators of the phenotype [20, 21].

*ANOS1* and *FGFR1* genes are all well established in the pathogenesis of CHH and have been extensively studied in many reported cohorts [20, 22–24]. Monogenic loss-of-function mutations of these major genes account together for up to 20% of KS cases, being the most common genetic causes of isolated GnRH deficiency. Due to rarity and heterogenicity of the condition the mutational spectrum, even in classical CHH genes, have yet to be fully characterized. To address this issue we screened for *ANOS1* and *FGFR1* variants in a cohort of 47 unrelated CHH subjects using targeted panel sequencing. Here we report novel variants identified in KS and nCHH patients.

## Methods

### Patients

A total of 47 unrelated patients (25 nCHH and 22 KS, including 31 men and 16 women) were studied by targeted Next Generation Sequencing (NGS). They were referred to the Department of Genetics for participation in genetic studies based on diagnosis of CHH. The diagnostic criteria included: clinical symptoms (absent or markedly delayed puberty, infertility, decreased libido), low testosterone/estradiol level with low or normal FSH and LH levels, no signs of other anterior pituitary dysfunction and no abnormalities of hypothalamic areas on radiological imaging. In case of Kallmann syndrome anosmia was assessed using formal testing or where unavailable by history only.

### Custom panel sequencing

Genomic DNAs from 47 patients were automatically extracted from peripheral blood leucocytes using the MagCore Genomic DNA Whole Blood Kit (RBC Bioscience), according to the manufacturer’s instructions. A custom panel (Illumina) was designed to capture CHH genes of interest, both previously associated with the condition and candidate genes. The 51 genes including *ANOS1* and *FGFR1* were selected based on literature searches (pubmed, OMIM). The panel covered all exons and intron/exon boundaries.

Probes for the targeted regions were designed using Illumina Design Studio the web-based software providing the 99% sequencing coverage of 1070 amplicons with an average length of 175 bp (2 × 150 base pair read length in paired-end mode) for the MiniSeq sequencer.

Libraries were prepared using TruSeq Custom Amplicon Low Input Library Prep Kit according to the manufacturer’s protocol (Illumina). All DNA samples were quantified and diluted to concentration of 10 ng/μl. After hybridisation, extension and ligation of oligos specific to the regions of interest, the libraries were barcoded, amplified, finally normalized, pooled and loaded into the cartridge (Illumina MiniSeq High Output Kit, 300 cycles). The PhiX library was combined with a prepared library and used as a sequencing control. Sequencing was performed on the MiniSeq platform (Illumina).

### Validation by sanger sequencing

Sanger sequencing as the gold standard to confirm nucleotide changes identified by NGS was used to verify selected class 3, 4 and 5 variants. Primers were designed to anneal upstream and downstream of DNA regions containing selected mutations of the *ANOS1* and *FGFR1* genes. After PCR amplification, products were sequenced using 3500 Genetic Analyzer (Thermo Fisher Scientific). Data were compared to the published *ANOS1* and *FGFR1* gene sequences NM_000216.2 and NM_001174067.1, respectively.

### Bioinformatical analysis

MiniSeq built-in software provided NGS data pre-processing. Quality control of raw read data was checked by FastQC and primer sequences were removed with Trimmomatic software. The fastq files were mapped to sequences in dedicated manifest files based on human reference genome (version GRCh37) with Isis Smith-Waterman-Gotoh 2.6.22.2. The aligned SAM file was processed with SAMtools to make a BAM file which was cleared of low quality mapped and duplicate reads (Picard tools). The read depth and coverage of BAM files were calculated for each region and each gene exon using BEDtools. Variants which did not comply with the requirements presented below were rejected from further analysis: population frequency < 2% (ExAC and 1000 Genomes database), read depth < 30 and alternative read depth < 10%. The variant calling was performed using the Isaac Variant Caller 2.1.4.2. Variants were annotated with Illumina BaseSpace Annotation Engine. Several prediction programs (PolyPhen, SIFT, NNSplice and MutationTaster, DANN, LRT, PROVEAN, dbNSFP.FATHMM, MetaLR, MetaSVM and MutationAssessor) were used to prioritize gene variants.

Variants were also evaluated for conservation across species using GERP and UniProt [25]. All variants were checked/searched in public databases: the ExAC, 1000 Genomes Project, The Genome Aggregation Database (gnomAD), Exome Sequencing Project, as well as HGMD and ClinVar.

UniProt alignment (Clustal Omega) was used to generate alignments between multiple sequences and analyse conservation across species of region of interests.

Criteria for variant classification and pathogenicity were used according to recommendations published elsewhere [26, 27].

All novel variants and those reported in CHH patients for the first time were submitted to ClinVar.

## Results

Targeted NGS in 47 unrelated patients, revealed two *ANOS1* and four *FGFR1* mutations in six subjects, of which five are novel and one was previously reported in CHH.

### *ANOS1* variants

Sequence analysis of the entire coding region of *ANOS1*, including exon–intron boundary regions, revealed two different hemizygous mutations: a single base pair deletion c.313delT and a single-base transition c.773G > A (Fig. 1).

A single base pair deletion c.313delT in exon 3 of *ANOS1* gene was identified in a KS male patient. The mutation results in a frameshift and a premature stop codon (p.Cys105ValfsTer13). The variant was not previously reported in any of the population variant databases including the ExAC, 1000 Genomes Project, The Genome Aggregation Database (gnomAD) nor in Exome Sequencing Project. Moreover, HGMD and ClinVar show no records for *ANOS1* c.313delT mutation. The variant was qualified as disease causing by MutationTaster and GERP. Based on ACMG–AMP criteria it was assigned to class 4, likely pathogenic. The variant was submitted to ClinVar and was assigned with accession number, SCV000996496.

Patient harbouring the mutation was diagnosed with KS at the age of 16 due to delayed puberty and anosmia. He was also reported to have bilateral synkinesia.

A male patient carrying a single-base transition c.773G > A was diagnosed with Kallman Syndrome at the age of 15 based on delayed puberty, hyposmia, unilateral cryptorchidism and laboratory test results. The c.773G > A transition replaces the normal corresponding codon (258) in exon 6 with a TGA stop codon (p.Trp258*). The variant was predicted as deleterious by PolyPhen, DANN, GERP, LRT and MutationTaster. Furthermore, it was not found in any of the population variant databases including the ExAC, 1000 Genomes Project, The Genome Aggregation Database (gnomAD) nor in Exome Sequencing Project. This nonsense mutation was previously reported in a family with 4 KS subjects in 2 generations [28]. Following ACMG–AMP recommendations the *ANOS1* c.773G > A variant was classified as class 5, pathogenic.

### *FGFR1* variants

Four heterozygous *FGFR1* variants were identified in studied cohort of CHH patients, none of which has been previously reported (Fig. 2).

A male carrying the 15 bp deletion c.374_388delTGCCCGCAGACTCCG in exon 4 (p.Val125_Ser129del) was diagnosed with Kallmann Syndrome at the age of 16 based on clinical symptoms and laboratory test results. He presented with underdeveloped male genitalia and anosmia, no other non-reproductive phenotype features were identified. This in-frame mutation is predicted to cause a deletion of five residues in the FGFR-1 protein. The mutation was classified as pathogenic by MutationTaster. No record on c.374_388delTGCCCGCAGACTCCG was found in any of the most-commonly used population variant databases. According to accepted criteria it was labelled as class 4, likely pathogenic. The variant was submitted to ClinVar and was assigned with accession number, SCV000996497.

All three identified missense mutations are novel and placed within *FGFR1* hot spots in exon 4, 15 and 17 encoding functionally important domains. The male patient harbouring c.331C > T has isolated hypogonadotropic hypogonadism with normal sense of smell. Variants c.1964 T > C and c.2167G > A were found in female patients diagnosed with Kallmann Syndrome. None of these variants were earlier reported in the ExAC, 1000 Genomes Project, The Genome Aggregation Database (gnomAD), Exome Sequencing Project. Moreover, both HGMD and ClinVar show no records.

Mutation c.331C > T localised in exon 4 is predicted to result in arginine to cysteine substitution (p.Arg111Cys). Prediction programs (PolyPhen, DANN, FATHMM-MKL, LRT, MutationAssessor, MutationTaster, PROVEAN, SIFT) classify the variant as pathogenic. UniProt alignment indicate that the Arg111 is a conserved residue. Based on ACMG–AMP 2017 recommendations mutation was assigned to class 4, likely pathogenic. The variant is novel and has been submitted to ClinVar, accession number SCV000996495.

Another *FGFR1* missense variant c.1964 T > C (p.Leu655Pro) was identified in a female with KS referred to genetic counselling because of delayed puberty and anosmia. The variant is localised in exon 15 within the sequence encoding for tyrosine kinase domain. The domain is functionally important and its DNA sequence is a known mutational hot-spot. UniProt alignment indicate that the Leu655 is a highly conserved residue. According to UniProt 95.7% of variants within the domain sequence is pathogenic. The variant was predicted as deleterious by PolyPhen, SIFT, DANN, GERP, LRT and MutationTaster. It shows no records in HGMD nor in ClinVar. Following ACMG–AMP recommendations the *FGFR1* c.1964 T > C variant was classified as class 4, likely pathogenic. The variant is novel and has been submitted to ClinVar, accession number SCV000996494.

Second female KS patient was found to have heterozygous c.2167G > A (p.Glu723Lys) variant in exon 17 of *FGFR1* gene. The substitution was identified in tyrosine kinase domain, a recognised hot-spot region. Glu723 is a highly conserved residue across species. All used prediction programmes classified the variant as pathogenic. Based on ACMG–AMP 2017 recommendations mutation was assigned to class 4, likely pathogenic. The variant is novel and has been submitted to ClinVar.

## Discussion

We performed panel NGS in a cohort of CHH probands (*n* = 47). Here we report novel variants in two well-known classical CHH genes: *ANOS1* and *FGFR1*.

*ANOS1*, formerly called *KAL1* gene encodes for anosmin-1 and is responsible for the X-linked form of Kallmann Syndrome [6]. ANOS1 loss of function due to mutations such as whole gene or intragenic deletions, frameshift, nonsense or missense mutations have been described and contribute to KS phenotype in 5–10% of cases [16]. Here we report two hemizygous *ANOS1* variants: a novel single base pair deletion c.313delT and previously reported single-base transition c.773G > A. *ANOS1* c.773G > A was first described by HardelinJ.P et al. in a single family with 4 subjects in 2 generations harbouring the mutation and presenting KS phenotype [28]. Our report of the same variant in unrelated patient with consistent KS characteristics strongly supports the genotype-phenotype correlation in the lack of functional data. Based on ACMG–AMP recommendations the variant was classified as class 5, pathogenic. The novel *ANOS1*c.313delT variant is also predicted to be highly deleterious as similar to nonsense mutations deletions causing frameshifts result in premature stop codons leading to the production of truncated proteins or to nonsense-mediated mRNA decay [29]. Loss of function is therefore highly probable in those with a reported cases of single base pair deletion in the coding sequence of *ANOS1*. Following ACMG–AMP recommendations the variant was classified as class 4, likely pathogenic and submitted to ClinVar.

*FGFR1* is one of the CHH-linked genes that is involved in both normosmic CHH and Kallmann Syndrome [17]. The prevalence of its mutations is ~ 6% in CHH compared to the 3–6% reported rate of *ANOS1* mutations [16]. The majority of FGFR1 mutations identified in CHH subjects are single amino acid substitutions located in the immunoglobulin-like domains or tyrosine kinase domains [13, 17, 30].

Here we report three novel missense variants of *FGFR1* predicted to result in the single amino acid substitutions c.331C > T (p.R111C), c.1964 T > C (p.L655P) and c.2167G > A (p.E723K). The Arg111Cys substitution in the first immunoglobulin-like domain removes a conserved arginine. It was predicted as damaging by several dedicated software tools and based on ACMG–AMP 2017 recommendations assigned to class 4, likely pathogenic. Other mutations p.G97D, p.Y99C and p.V102I localised within the IgI domain in close proximity to p.R111C have been reported in CHH [15, 31]. This cluster of missense mutations in the IgI domain strongly supports its importance in receptor function.

Both Leu655 and Glu723 are localised in the tyrosine kinase domain TK2. All used prediction programmes classified Leu655Pro and Glu723 Lys variants as pathogenic. Based on ACMG–AMP 2017 recommendations mutations were assigned to class 4, likely pathogenic. Available data on other substitutions in FGFR-1 TK domains predict a decrease or inhibition of kinase activity by disrupting the receptor conformation (Ile538Val, Asn724Lys, and Gly703Arg) and/or altering the normal pattern of the domain’s phosphorylation (Ala520Thr, Gly703Ser, Pro722Ser, Pro745Ser, and Pro772Ser) [17, 30]. We assume that similarly Leu655Pro and Glu723 Lys substitutions may impact the TK2 domain conformation and thus its enzymatic activity.

Further supportive evidence that our *FGFR1* missense mutations are deleterious comes from UniProt alignment (Fig. 2d), which indicates that all three mutations involve highly conserved AA residues among known species and therefore are not likely to be tolerated by their observed substitutions.

In addition to the three missense variants we identified a novel 15 bp deletion c.374_388delTGCCCGCAGACTCCG in exon 4 of *FGFR1*. The identified in-frame deletion is located in a small region of the IgI-IgII interdomain rich in acid residues. This acid box provides an autoinhibition mechanism and prevent FGF-independent activation of FGFR by heparan sulphate proteoglycans [32]. It is normally bound to the heparan sulphate binding basic region on IgII, thereby competing with glycosaminoglycans for FGFR1-binding. A missense mutation D129A mapping in this region had previously been reported in a KS patient, yet its functional consequence in the context of a loss-of-function mutations, was unclear [31]. As an identified 15 bp deletion is predicted to remove 5 AA residues p.V125_S129del it is likely to result in conformational changes in addition to possible interference with IgII binding. According to accepted criteria the novel variant was labelled as class 4, likely pathogenic.

The study has several limitations. There are no data available on identified rare variants that could potentially explain their functional phenotypic effect and provide a final proof for the mutation pathogenicity. We were able to collect parental samples in the majority of paediatric patients, in contrast many adult patients declined family testing. Finally, the studied cohort of 47 individuals is relatively small compared to multicentre, often international projects enrolling a large number of CHH patients [24, 33].

Considering our above findings, protein structural predictions, the rarity of reported variants and amino acid conservation in case of missense substitutions all provide support that these mutations are highly likely to be deleterious. *ANOS1* mRNA nonsense-mediated decay, ANOS1 protein truncation or disruption of important evolutionary conserved FGFR1 domains, all indicate detrimental effects on these proteins function.

## Conclusions

The findings from the present study expand the mutational spectrum of *ANOS1* and *FGFR1* in hypogonadotropic hypogonadism. Further analyses of known and candidate genes implicated in congenital hypogonadotropic hypogonadism will likely continue to support the genetic complexity of the disorder. The knowledge of the full genetic spectrum of CHH is increasingly important in order to be able to deliver the best personalised medical care to our patients.

## Data Availability

The datasets used and/or analysed during the current study are available from the corresponding author on reasonable request.

## Abbreviations

FSH: Follicle-stimulating hormone
GnRH: Gonadotropin-releasing hormone
KS: Kallmann Syndrome
LH: Luteinizing hormone
nCHH: Normosmic congenital hypogonadotropic hypogonadism
NGS: Next generation sequencing
